# Knowledge, Attitudes, and Food Safety Practices of Informal Market Maize Grain Vendors and Consumers in Meru County, Kenya

**DOI:** 10.1155/ijfo/6592430

**Published:** 2024-11-21

**Authors:** Cherotich Cheruiyot, Michael W. Okoth, George O. Abong', Sarah W. Kariuki

**Affiliations:** ^1^Department of Food Science Nutrition and Technology, University of Nairobi, PO Box 30197-00100, Nairobi, Kenya; ^2^Department of Molecular Biology and Genetics, International Maize and Wheat Improvement Center (CIMMYT), PO Box 1041-00621, Nairobi, Kenya

**Keywords:** aflatoxin contamination, awareness, quality techniques, subcounties

## Abstract

Aflatoxin contamination in food poses a significant health risk, especially in low- and middle-income nations. While there is a need to increase food safety, there is a lack of aflatoxin-related sociodemographic factors, knowledge, attitudes, and practices (KAPs) among aflatoxin-risk populations. The study is aimed at collecting information from the vendors and consumers of maize grains to investigate their sociodemographic factors and KAPs of aflatoxin contaminations in Kenya's small- and medium-sized informal markets. A cross-sectional survey using a simple random sampling approach was conducted from July 2022 to August 2022 in Meru County, Kenya. Vendors and consumers of maize grain markets were interviewed using face-to-face interviews and semistructured questionnaires. Females were the predominant gender among the maize grain vendor (82.7%) and consumer (83.72%) participants. Most vendors (95.7%) and consumers (94.2%) have formal education. Almost all vendors (96.2%) and consumers (95.3%) check for maize selling and purchasing quality. The proportion of visibly mouldy grains (25.5%, 29.0%), moisture level (20.2%, 12.9%), and size of grains (11.4%, 14.8%) were the commonest quality dimensions checked by vendors and consumers, respectively. Most vendors (86.8%) and consumers (70.9%) have heard about aflatoxin, but only 48.2% of the vendors and 52.1% of the consumers were aware and could mention the correct aflatoxin health effects. Vendors' and consumers' KAPs on aflatoxin varied significantly (*p* < 0.05) across subcounties, education levels, genders, and ages. Promoting public awareness, building capacity, and implementing aflatoxin-related policies at all levels are crucial to ensure food safety.

## 1. Introduction

Consumption of unsafe food is a major cause of foodborne diseases which are main health concerns that impede socioeconomic development. In 2010, 31 food safety hazards were associated with 600 million illnesses and 420,000 deaths worldwide according to the World Health Organization [1]. According to the World Bank report of 2019 [2], foodborne diseases have led to total productivity loss in low- and middle-income countries (LMICs) estimated to be $95.2 billion annually, while $15 billion is required annually to treat foodborne illnesses. Most cases of foodborne disease have been reported in LMICs, especially countries in Sub-Saharan Africa (SSA) [1, 3].

Mycotoxins are toxic secondary metabolites and a major food safety hazard produced by certain species of fungi that continue to draw global interest due to their substantial economic value and impact on human health, animal production, and local and international trade [4]. Mycotoxins contaminate cereals, nuts, and fruits during pre- and postharvest periods [5]. In SSA, 40%–80% of farm produce were lost at the postharvest stage [6–8]. Per the Food and Agricultural Organization (FAO) [9], 25% of the world's produce is contaminated with mycotoxins at pre- and postharvest stages. Some of the major mycotoxins include aflatoxins, fumonisins, deoxynivalenol (DON), T-2 toxins, and zearalenone (ZEA) [10, 11]. Mycotoxins have hazardous health effects on consumers, which may be acute or chronic, depending on the aflatoxin concentration in the consumed products and the duration of exposure [12]. Large concentrations result in acute illness and mortality, typically due to liver cirrhosis, while chronic sublethal doses have nutritional and immunologic repercussions. Studies have shown that high ingestion of aflatoxin-contaminated food by humans for a long time can result in chronic health effects such as immune suppression [13, 14], high rates of illness, stunted growth in infants and children, esophageal cancer [15, 16], and liver cancer [17]. Chronic exposure has also been linked to other health effects such as mutagenicity, teratogenicity, estrogenicity, and cytotoxicity [18]. Aflatoxin–protein adducts have been mostly associated with acute intoxication [19]. Persistent exposure to aflatoxin through ingestion can cause gut inflammation, leading to enteropathy and the malabsorption of essential nutrients in the body [20, 21]. Ingestion of aflatoxin also increases the effect of other opportunistic diseases, for example, primary hepatocellular carcinoma (HCC) and rapid progression from HIV to AIDS [20]. Aflatoxicosis may result in mortality [12, 22, 23].

Cereals, particularly maize, are widely consumed in many SSA countries and are prone to aflatoxin contamination [24]. Poor storage of maize grains especially in high-temperature and high-humidity conditions can exacerbate the growth of aflatoxin-producing fungi [12, 25]. In light of Africa's subsistence-based agricultural systems, regulations have not been implemented at the household level [11]. In SSA, cereals are traded between different regions within the country where there is no control measure to curb the potential spread of aflatoxin. Food safety standards may vary from country to country [26]. Traders have reported tremendous losses due to latent symptoms of aflatoxins expressed late in the market and fresh infections of maize grains under poor storage conditions at various informal markets. Reportedly, aflatoxin contaminations have arisen in eastern Kenya, leading to the destruction of infected maize grains and huge losses of human life for those who consumed infected maize [27].

LMICs, including Kenya, have a higher incidence of food safety hazards. In these countries, informal markets have a high likelihood of aflatoxin prevalence due to challenges such as limited resources for proper agricultural practices, weaker formal institutions for monitoring and prevention, inadequate knowledge about aflatoxin, profit-driven behaviors, and minimal regulatory oversight and implementation [28–33]. This shows that maize of low quality is either used for personal consumption or traded locally in the informal markets.

A previous study in Kenya documented a strong correlation between the quality of maize in terms of the outer layer of the kernels (an observable trait) and the level of aflatoxin (an unobservable attribute) contamination [34]. The detection of aflatoxin (an unobservable attribute) in food products is done using specialized tests that are expensive and not accessible to many stakeholders across the maize value chain, especially in the informal village markets.

The maize value chain is often characterized as long and fragmented with many actors in many SSA countries [35]. For instance, these actors include farmers, maize traders, feed millers (maize and final feed) and retailers of maize-based products, and consumers [35]. Therefore, the very long supply chains often create avenues for increased risks of aflatoxin contamination during maize production, handling, and storage [35]. Studies have already reported high levels of aflatoxin upstream of the maize value chain [35, 36]. To limit the risks of aflatoxin exposure to consumers of maize grains, there is a need to assess the knowledge, attitudes, and practices (KAPs) of all actors in the value chain. Previous studies have demonstrated KAPs among the farmers representing one node of the value chain [37, 38]. However, there is scant information on KAPs among the traders (vendors) and consumers which is needed to design effective aflatoxin management measures across all value chains, especially in the informal markets. Formal markets are confined to regulated frameworks where sellers are free to disclose their prices and locations; usually, they serve larger-scale corporate transactions and follow strict quality and consumer protection standards. On the other hand, informal markets operate in unregulated spaces and deal with smaller-scale transactions with little oversight where the vendors negotiate bilaterally to keep their identities hidden from tax authorities [39]. Therefore, this study was conducted to provide insight into vendors' and consumers' sociodemographics and KAPs on aflatoxin-contaminated maize grains, health concerns, and methods of reducing aflatoxin contamination or exposure in the informal markets. This study's result will stimulate awareness among vendors and consumers regarding aflatoxin prevalence and how to reduce the consumption of aflatoxin-contaminated maize by using observable attributes that are cheap and easily affordable, especially when selecting maize grains for use. Therefore, reducing aflatoxin contamination in maize grains can improve food safety and the overall health of consumers as well as enhance market ingress [40]. The results will help stimulate the formulation of policies regarding aflatoxin management in the informal markets since consumers will demand better-quality maize grain during purchase.

## 2. Materials and Methods

### 2.1. Study Area

**Figure 1:**
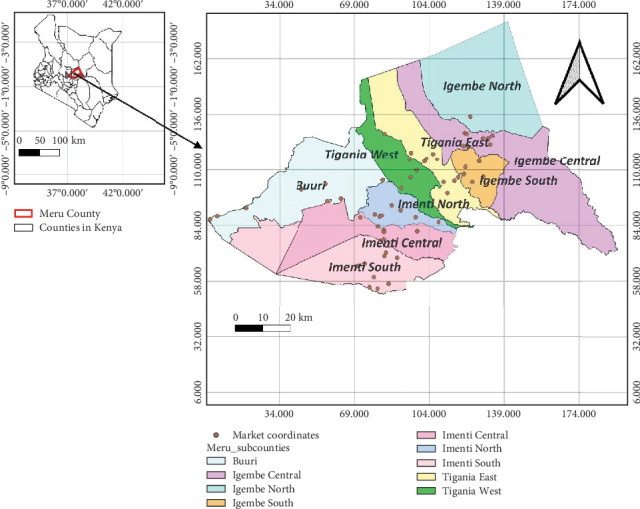
Map of the study area showing Meru County, subcounties, and study points (markets) in Kenya.

Meru County is located in the eastern region of Kenya (Figure 1). It comprises nine subcounties Buuri, Imenti North, Imenti Central, Imenti South, Igembe North, Igembe Central, Igembe South, Tigania East, and Tigania West. The county occupies an area of 6936 km^2^ with seven subagroecological zones varying from the highlands (central and north parts) and midlands to lowlands [41]. The county generally receives rainfall of 380 mm in the lowlands and 2500 mm in the highlands and lies between 300 and 5199 m (above sea level) [42]. The area also experiences bimodal rainfall with the long rains occurring from mid-March to May and short rains from October to December [43]. The Meru tribe primarily inhabits the area, and the population size is estimated at 1,545,714 persons according to 2019 census data [44]. The main activity is crop production, and the key crops grown include potatoes, maize, sorghum, millet, and wheat. The county is among Kenya's hotspots of aflatoxin contamination [45].

### 2.2. Study Design and Period

We conducted a cross-sectional study using a random sampling approach where each maize grain vendor and consumer had an equal chance of being selected. The study covered most maize grain informal markets in Meru County. The study was conducted from July 2022 to August 2022. Before the interview, the enumerators sought permission from the vendors and consumers of maize grains.

### 2.3. Study Population

A survey was conducted by administering semistructured questionnaires to vendors and consumers of maize grains. Nine subcounties in Meru County, namely, Buuri, Igembe Central, Igembe South, Igembe North, Imenti South, Imenti North, Imenti Central, Tigania East, and Tigania West were involved in the study. Small- to medium-sized informal markets were purposefully selected. Large markets were avoided to ensure size uniformity of the study markets in terms of the number of maize grain vendors and consumers. Before the selection of study markets, a list of all informal markets was obtained from the office of trade in Meru County during the previsit to the area. Details of the main contact persons at market levels were also provided. A short phone call survey was conducted to interview the contact persons. The information sought included validation of the market names, main market days, and estimated number of retail vendors in each market. The markets without a reachable contact person or any maize traders were excluded. The study included 90 markets in total.

### 2.4. Eligibility Criteria

Vendors and consumers of maize grain who consented and are above 18 years of age in small- to medium-sized informal markets were included in this study while those who did not consent were excluded.

### 2.5. Study Variables

In this study, the dependent variables were KAPs related to aflatoxin-safe maize grains. The independent variables were sociodemographic factors (age, gender, and education status), market type, and subcounty.

### 2.6. Sample Size Determination

The local authorities provided the estimated population of vendors in small- to medium-sized informal markets within the nine subcounties of Meru County. With the assistance of county authorities, the subcounty's administrative boundaries were established. The sample size of vendors was summed to subcounty levels. In each subcounty, the size of sampled and interviewed vendors was distributed according to the proportion of vendors in each market. The sample size of the vendors as respondents in nine subcounties in Meru County was computed using Cochran's formula [46]:

$$\begin{array}{cc} \; & n_{\circ }=\frac{Z^{2}pq}{d^{2}}\\ n_{1}=\frac{n_{\circ }}{\left(1+n_{\circ }/N\right)} \end{array}$$

where *n*_∘_ is the desired sample size, *n*_1_ is the corrected sample size, *N* is the population size, *z* is 1.96 (confidence level at 95%), *p* is the proportion of 0.9, *q* is 1 − *p*, and *d* is the level of precision (0.05).

Cochran's formula established the sample size of vendors interviewed in the nine subcounties, Meru County (Table 1).

Although many consumers visited the market to purchase the maize grains from the vendors, most could not consent to participate in the interview with the majority citing time constrain. Therefore, about 3–5 consumers who visited each market to purchase maize grains from the interviewed vendors were randomly selected and interviewed. A total of 452 traders and 86 consumers of maize grains in all nine subcounties were selected for the study.

### 2.7. Data Collection

To assist with data gathering, 15 enumerators were trained. Upon arrival in each informal market, enumerators surveyed the market to ascertain the number of maize grain traders. Through the use of pretested sets of semistructured questionnaires, qualitative data on the demographics of the vendors and consumers as well as their KAPs on aflatoxin contamination of maize grains were collected. A blend of local language, Kiswahili, and English was used during the interviews. A data collection platform (SurveyCTO tool) was used in this study.

### 2.8. Association of Sociodemographic Profiles With Aflatoxin KAPs

To do this, the responses from the vendors and consumers were reviewed. The correct responses based on the facts and previous documentation were confirmed and scored (Tables 2 and 3).

### 2.9. Statistical Analysis

Data from the questionnaires were entered into Microsoft Excel. The data was checked by cross-tabulations for accuracy, and any discrepancy was corrected or removed. The data collected from the respondents were converted to frequencies (*n*) and percentages (%) for basic descriptive statistics. The chi-square test for independence was used to compare frequencies for KAP factors. Chi-square analysis was also used to test the association between sociodemographic factors and knowledge of aflatoxin contamination, attitudes, and practices toward the reduction of aflatoxin contamination in maize grains. A post hoc test was performed for independent variables with statistical significance. The significance of the association was ascertained at a *p* value less than 0.05 (95% confidence interval). Further, the correctness of responses was confirmed based on literature and converted into a percentage of respondents. This dataset was subjected to chi-square analysis.

### 2.10. Ethics Approval and Consent to Participate

Ethical clearance and permission were sought from the Amref Ethics and Scientific Review Committee (ESRC) with Protocol Identification Number ESRC PI 141/2022. Additionally, permission to conduct research in Meru County was provided by the National Commission for Science, Technology, and Innovation (NACOSTI) License No. NACOSTI/P/22/17001. Before any data was collected, each respondent was made aware of the purpose of the study and given the chance to give their oral consent. The interviews were conducted voluntarily and with consent. A very high degree of privacy and confidentiality was applied to the data that the respondents supplied. The vendors and consumers provided only the information needed for this investigation. Because of the COVID-19 epidemic during the survey, every study participant was provided with masks and hand sanitizer to use in addition to maintaining social distancing.

## 3. Results

### 3.1. Sociodemographic Characteristics of Study Vendors and Consumers

The demographical data is summarized in Table 4. A total of 452 vendors and 86 consumers were interviewed. The results showed that 131 (30.5%) of the vendors were 40–49 years old. One hundred and fifteen (26.7%) were aged between 30 and 39 years. The remaining 71 (16.7%), 58 (13.5%), and 55 (12.8%) of the vendors were aged between 18 and 29, 50 and 59, and above 60 years, respectively, while 24 (27.9%) of the consumers purchasing maize grains were aged between 30 and 39 and 22 (25.6%) were 18–29 years. The remaining 17 (19.8%), 14 (16.3%), and nine (10.5%) of the consumers were aged between 40 and 49, 50 and 59, and above 60 years, respectively.

Most (82.7%) of the vendors and most (83.7%) consumers of maize grain were female. Most vendors (47.1%) and consumers (53.5%) have completed primary school education while 4% of the vendors and 5% of the consumers had no formal education.

The majority (84.9%) of the consumers interviewed said they were maize farmers. Most (75.4%) of the vendors own kiosks or shops while 24.6% use open-air markets or erected temporary shades to sell their maize grains.

Most (22.8%) of the vendors interviewed came from Imenti South followed by Igembe Central (15.7%) and then Buuri (14.4%). Most (17.3%) of the consumers who purchased maize grains came from Igembe Central, followed by Tigania East (13.6%), Tigania West (12.3%), Buuri (12.3%), and Imenti South (12.3%).

### 3.2. Knowledge of Aflatoxin Contamination Among Maize Grain Vendors and Consumers

The level of knowledge about aflatoxin contamination among maize grain vendors and consumers is presented in Table 5. The findings revealed that many vendors (86.8%) and consumers (70.9%) had heard of aflatoxins. Stomach pain was the most commonly recognized symptom of consuming aflatoxin, reported by 48.2% of the vendors and 52.1% of the consumers. This shows that vendors and consumers' awareness of the danger of aflatoxin contamination was average. Other perceived signs of consuming aflatoxin included cancer, death, diarrhea, and increased vulnerability to other diseases. However, the least recognized symptoms among vendors and consumers were liver failure/jaundice, vomiting, bloating, headache, constipation, coughing, having yellow eyes, heart problems, kidney issues, loss of appetite, ulcers, stunted growth, lung problems, and memory loss. In addition, the study also looked at the quality dimensions assessed by the vendors and consumers when purchasing maize grains. It was found that the most checked quality parameters by vendors were the proportion of visibly mouldy grains, moisture level, the proportion of foreign matter in the maize, and the size of the grain. On the other hand, consumers mostly checked on the proportion of visibly mouldy grains, followed by the size of the grain, the proportion of foreign matter in the maize, moisture level, and the proportion of broken maize kernels.

#### 3.2.1. Maize Grain Quality Dimensions Assessed by the Vendors and Consumers

Table 6 presents the quality attributes checked by the vendors and consumers while purchasing maize grains. The most checked quality parameters by the vendors were the proportion of visibly mouldy grains (25.5%), moisture level (20.2%), the proportion of foreign matter in the maize (13.3%), and the size of the grain (11.4%), while the least (< 10%) checked quality dimensions were the proportion of broken maize kernels, insect infestation, aflatoxin levels, color change, presence of pesticides, maize variety, and weight of maize. Only 16.8% of the vendors mix different qualities of maize to improve or to get an average grade of maize grains. On the other hand, consumers mostly checked on the proportion of visibly mouldy grains (29.0%), followed by the size of the grain (14.8%), the proportion of foreign matter in the maize (14.2%), moisture level (12.9%), and the proportion of broken maize kernels (12.3%). The least (< 5.2%) checked parameters were insect infestation, aflatoxin levels, presence of pesticides, maize variety, and weight of maize. Most of the consumers had different ways of preparing and using maize grain; they mix with beans to make a famous food dubbed “Githeri” according to 36.4% of the consumers. Other consumers mill maize grains into flour to make “Ugali” (posho) (35.7%) or porridge (1.9%). Some consume purchased maize grains with corn whose testa (seedcoat) has been partially removed (“Muthokoi”) (22.1%), crushed maize (“Njenga”) (1.3%), and polished (“Muthikore”) (0.6%). Only a few (1.9%) acknowledge purchasing maize to feed their livestock.

### 3.3. Vendors' and Consumers' Attitudes Toward Aflatoxin-Contaminated Maize Grains

The majority of the vendors agreed (24.4%) and strongly agreed (66.7%) that they sell maize grains without broken kernels. However, most of the consumers disagreed (34.9%) and strongly disagreed (37.2%) that maize grains sold in the market are without broken kernels (Table 6). Regarding mouldy maize grains, the majority of the vendors strongly agreed (57.3%) and agreed (29.3%) that maize grains sold in various markets were mould-free. On the other hand, consumers disagreed (25.6%) and strongly disagreed (51.2%) that maize grains traded in the markets were not mouldy (Table 7). The biggest proportion of the vendors strongly agreed (41.2%) and agreed (35.1%) that they trade aflatoxin-free maize on most occasions while on the contrary consumers strongly disagreed (52.9%) and others disagreed (28.2%) that the maize sold in the markets are aflatoxin-free. Most consumers (41.9%) indicated that maize containing aflatoxin does not end up being consumed. Interestingly, the smaller proportion of consumers (cumulatively 18.6%) representing above 5/10 indicated their concern about maize with aflatoxin ending up being consumed (Table 7).

### 3.4. Practices Toward Minimizing Aflatoxin Contamination by Maize Grain Vendors and Consumers

#### 3.4.1. Vendors

There were significant differences (*p* < 0.05) in how vendors packaged and stored maize grains. Most vendors (87.3%) use gunny bags to package and store maize grains. Some (11.4%) use sisal bags while a minority (1.4%) of them use hermetic bags. The majority (83.8%) of the vendors stored maize grains on concrete floors (Table 8).

#### 3.4.2. Consumers

Just like vendors, most (64%) consumers always package and store maize grains in gunny bags, with a big portion of them (41.9% and 58.1%) never using hermetic bags and PICS bags, respectively. On a positive note, the majority (82.6%) indicated that they always maintain their maize grain under proper storage conditions. The study also showed that most of the consumers (35%) buy and dry their maize on open fields or by roadways. Only 2.3% of the consumers admitted that they dry their maize after purchase on bare ground without the canvas (Table 9).

### 3.5. Association Between Sociodemographic Characteristics and KAPs of Aflatoxin-Contaminated Maize Grains Among Vendors and Consumers

#### 3.5.1. Vendors

**Figure 2:**
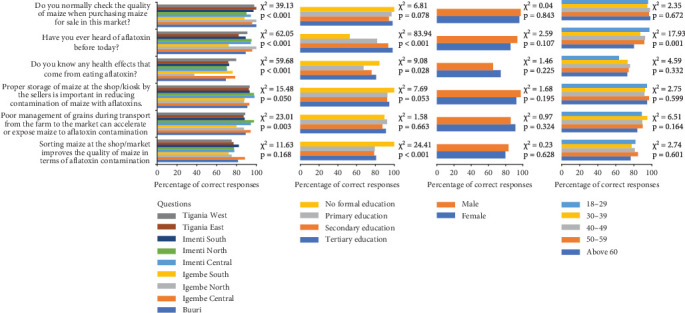
Association of sociodemographic factors with vendors' knowledge, attitudes, and practices on aflatoxin-safe maize grains.

Figure 2 illustrates an association between sociodemographic characteristics and KAPs of aflatoxin-contaminated maize grains among vendors.

##### 3.5.1.1. Subcounties

Most (72%–96%) vendors have heard about aflatoxin with Igembe South having the least (72%) number of respondents compared to the rest of the subcounties (82%–96%). Only Igembe North was the least (38%) knowledgeable about the health effects of aflatoxin compared to the rest of the subcounties (70%–80%). About 38%–80% of the vendors correctly mentioned the health effects of consuming aflatoxin-contaminated maize grains. Most (88%–98%) vendors believed properly storing maize grain at the shop/kiosk is important in reducing aflatoxin contamination. The majority (80%–88%) of the maize grain vendors agreed that proper management of maize grain during transportation from the farm to the marketplace reduces the exposure of the maize grains to aflatoxin contamination.

##### 3.5.1.2. Education Levels

Most (94%–100%) of the vendors checked for the quality of maize grains while purchasing for stocking and sale irrespective of their education status. Vendors with no formal education have least (53%) heard about aflatoxin followed by vendors who attained primary education level (82%) while the majority (93%, 98%) of the vendors with secondary education level and tertiary education level, respectively, have heard about aflatoxin. Most (87%–92%) vendors acknowledged that poor management of maize grains during transport from the farm to the market can accelerate or expose maize to aflatoxin contamination. Compared to vendors with tertiary education (81%), secondary education (78%), and primary education (79%), vendors with no formal education mostly (100%) acknowledged that sorting maize at the shop/market improves the quality of maize in terms of aflatoxin contamination.

##### 3.5.1.3. Gender

There was no significant variation (*p* > 0.05) between the male and female vendors regarding KAPs of aflatoxin-contaminated maize grains across the subcounties.

##### 3.5.1.4. Age

Apart from having heard about aflatoxin, there was no significant variation (*p* > 0.05) among the age groups in their response regarding KAPs of aflatoxin-contaminated maize grains. Vendors within the age group of 18–29 years (97%) have mostly heard about aflatoxin followed by vendors within the age groups of 40–49 years (92%) and 50–59 years (91%) while vendors with age above 60 years have least heard about aflatoxin.

#### 3.5.2. Consumers

**Figure 3:**
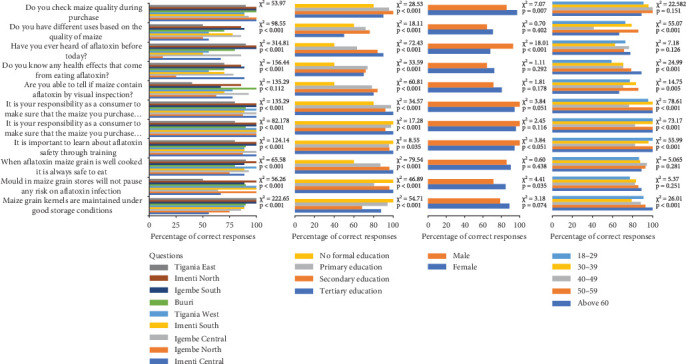
Association of sociodemographic factors with consumers' knowledge, attitudes, and practices on aflatoxin-safe maize grains.

Figure 3 illustrates an association between sociodemographic characteristics and KAPs of aflatoxin-contaminated maize grains among consumers.

##### 3.5.2.1. Subcounties

Consumers' knowledge regarding aflatoxin awareness, health effects, storage of maize grains, practice of checking maize quality, management of maize during transportation, and sorting of maize in terms of aflatoxin varied significantly (*p* < 0.05) across the nine subcounties. All interviewed consumers in Imenti Central, Igembe North, Buuri, Igembe South, and Imenti North acknowledged (100%) that they normally check for the quality of maize they purchase for their consumption followed by consumers (89%–93%) at Igembe Central, Imenti South, Tigania West, and Tigania East. Igembe South and Imenti South comprise the greatest number of consumers (100%) who have heard about aflatoxin followed by Tigania East (90%), Igembe Central (86%), Buuri (80%), Imenti Central (67%), Tigania West (56%), and Imenti South (50%) while Igembe North had the least proportion of respondents who have heard about aflatoxin (13%). Only Igembe North (25%) followed by Tigania West (50%) had the lowest proportion of consumers who are knowledgeable about the health effects of aflatoxin compared to the rest of the subcounties (70%–90%).

Buuri (100%) and Igembe Central (93%) comprised subcounties whose consumers visually inspect maize grains for potential aflatoxin contamination. These were followed by Imenti North (86%), Imenti Central (78%), Tigania West (78%), Imenti South (70%), Igembe South (67%), and Igembe North (63%), while Tigania East had the least proportion (40%) of consumers who can visually inspect maize grains for aflatoxins. About 75%–100% of the consumers in all subcounties believed that, when aflatoxin maize grain is well cooked, it is not always safe to eat; it is important to learn about aflatoxin safety through training; and it is their responsibility to make sure that the maize they purchase is safe from aflatoxin contamination and is of good quality. Most (89%–100%) consumers from Igembe North, Imenti South, Tigania West, Buuri, Igembe South, and Imenti North reported that moulds in maize grain stores will not pose any risk of aflatoxin infection, while the least number of consumers with the same attitude was reported in Imenti Central (67%), Igembe Central (64%), and Tigania East (50%). Unlike the rest of the subcounties (86%–100%), Imenti Central and Igembe North had the least number of consumers 56% and 75%, respectively, who agreed that maize grain kernels should be maintained under good storage conditions.

##### 3.5.2.2. Education Levels

Consumers' responses regarding the KAPs varied significantly (*p* < 0.05) based on education levels. Most (90%–100%) of the consumers with formal education checked maize quality during purchase while 80% of the consumers had no formal education. Most (63%–90%) consumers have heard about aflatoxin before, know the health effects associated with eating aflatoxin-contaminated maize (70%–74%), and can tell if maize contains aflatoxin by visual inspection (78%–84%). Consumers with no formal education (40%) have heard of aflatoxin before and know the health effects associated with eating aflatoxin-contaminated maize grains. Most consumers (60%–100%) agreed that it is their responsibility to make sure that the maize grains they purchase are of good quality and safe from aflatoxin contamination; it is important to learn about aflatoxin safety through training; maize grain kernels are maintained under good storage conditions; mould in maize grain stores will not pose any risk on aflatoxin infection; and when aflatoxin maize grain is well cooked, it is not always safe to eat. However, there were significant variations (*p* < 0.05) in education levels.

##### 3.5.2.3. Gender

More females (97%) than males (86%) check the quality of maize during purchasing. Likewise, more females (89%) than males (79%) believed that moulds in maize grain stores do not pose any risk of aflatoxin infection. In contrast, more males (93%) than females (68%) have heard about aflatoxin before. However, there was no statistical variation (*p* > 0.05) between males and females in terms of knowledge of health effects associated with aflatoxin, ability to visually identify insect maize grains for aflatoxins, beliefs on purchasing good quality maize, aflatoxin-free maize grains, learning about aflatoxin safety during training, and maintaining maize grains in good storage conditions.

##### 3.5.2.4. Age

There were no significant variations (*p* > 0.05) among the age groups in terms of the ability to check maize quality during purchase, having heard about aflatoxin before, beliefs on reduction of aflatoxin levels through cooking, and the presence of mould in maize grain store posing no risks of aflatoxin contamination. However, there was significant variation (*p* < 0.05) with no obvious trend among age groups in terms of knowledge of health effects that come from eating aflatoxin, ability to tell if maize contains aflatoxin by visual inspection, beliefs in making sure that the maize they purchase is of good quality, safe from aflatoxin contamination, and importance of learning about aflatoxin safety through training.

## 4. Discussion

We examined the sociodemographic factors and knowledge, attitudes, and food safety practices regarding aflatoxin contamination of maize grain among vendors and consumers in the informal markets of Meru County. Females were the predominant gender among the maize grain vendors and consumers purchasing maize grains. This phenomenon has previously been reported by De Vletter and Polana [47] showing that the purchase and wholesaling of maize grains at the informal markets in southern Mozambique were predominated by women. Another study by Kamano et al. [37] among vendors in Makueni County, Kenya, showed that 45% of the responders were male and 54.55% were female. On the contrary, a study conducted in Baringo County, Kenya, by Kamano et al. [48] confirmed that most of the maize grain farmers were men (67.27%), while 32.73% were women.

Overall, knowledge of vendors and consumers regarding aflatoxin contamination was based on general awareness, capacity to state the signs and symptoms of consuming unsafe levels of aflatoxins, ability to visually detect maize grains contaminated with aflatoxins, and conditions that favor or are associated with aflatoxin contamination. Our findings showed that more educated respondents were more knowledgeable about aflatoxin awareness, health effects, and visual inspection of maize quality. A study by Suleiman, Rosentrater, and Chove [6] showed that education positively correlates with awareness of mycotoxins. The results showed adequate knowledge levels in Kenya compared to other countries in East Africa. In contrast, the findings in this study contradicted with results in a study by Suleiman, Rosentrater, and Chove [6], showing that most Tanzanian farmers and traders did not know about aflatoxin contamination.

The respondents mostly checked for the presence of moulds and moisture levels, while the least checked quality parameters were the proportion of broken kernels, discolored grains, and the level of insect damage. These parameters constitute critical observable signs of aflatoxin contamination in maize grain relied on by most vendors and consumers in the informal markets [29]. Few vendors and consumers also checked for aflatoxin levels (an unobservable attribute) on the grains they purchased. High moisture levels, a large proportion of broken maize kernels, high insect infestations, and poor storage may contribute to aflatoxin incidences [49]. Most respondents use traditional methods such as hands and biting as the commonest methods to verify grain dryness of maize grains. However, these methods are unreliable and could result in grains being stored with a high residual moisture content, encouraging fungal growth, which can aggravate aflatoxin contamination [50].

Most consumers used the maize grains they purchased for the preparation of foods mainly “Githeri,” “Ugali,” and “Muthokoi.” These kinds of food preparation have been reported to reduce aflatoxin levels. For instance, a study done by Mutungi et al. [51] showed that preparation of “Muthokoi” could decrease aflatoxin levels by 46.6% during dehulling, 72% during soaking of maize grains in sodium hypochlorite or ammonium persulfate for 6–14 h, and 80%–93% during boiling at 98°C for 150 min. Matumba et al. [52] also found that half of the population believed basic cooking could destroy toxins. On the contrary, some studies have shown that basic cooking can reduce but not eliminate mycotoxins, because of the heat-resistant nature of some mycotoxins; hence, processing techniques may be insufficient [53, 54]. However, some other studies have shown drying maize meal at 190°C for 60 min and 220°C for 25 min, respectively, can result in a 60%–100% reduction of aflatoxin [55]. These discrepancies should be related to a type of mycotoxin and levels of contamination in food [11].

Although many consumers cited that they have different uses for maize grains based on their quality, the use of spoiled grains especially as animal feed and alcohol production may increase exposure to aflatoxins [56–58]. Most consumers did not dispose of maize of poor quality or contaminated maize but used it in various ways while some vendors mixed it with clean maize to improve the overall quality. However, this still predisposes the consumers to aflatoxins. Blending poor-quality with high-quality grains is a common practice that has previously been reported in Kenya by Kamano et al. [37].

Most respondents sorted the maize grains to improve their quality regarding aflatoxin contamination. Sorting is important and can reduce exposure to mycotoxin contamination from 80% to 40% [59]. Sorting techniques may include separating discolored or mouldy kernels by handpicking, winnowing, washing, crushing, and dehulling of maize grains which can reduce mycotoxins before storage [8, 60–62].

Drying is considered the most critical postharvest management practice for reducing moisture levels in cereal grains [60]. Between 19% and 25% of cereals' moisture levels favors germination of grains, insect infestation and reproduction, and most importantly the growth of aflatoxin-causing fungus [62]. Manu et al. [49] recommend that 13% and below moisture levels significantly reduce aflatoxin contaminations. In the context of SSA, substandard methods of drying maize grains before, during, and after harvesting do not significantly reduce aflatoxin contamination [63].

Most vendors packaged their maize grains in gunny bags and stored them on concrete floors. Several aflatoxin management practices such as sorting, proper storage techniques, and having different uses for grains exhibiting signs of spoilage can minimize human exposure to aflatoxin through consumption [48]. Aflatoxin-producing fungi are favored by broken grains, insect infestation, high humidity, high temperatures, greater concentrations of CO_2_, drought stress, and poor pre- and postharvest practices [53, 64, 65]. These conditions enable fungal growth and mycotoxin production, making SSA a vulnerable region to the mycotoxin contamination of crops.

About 20%–30% of crop loss in SSA occurs during storage [66, 67]. It is worth knowing that poor storage of crop products affects the quality and predisposes the product to mycotoxin contamination [68]. Traditional storage methods remain widely used by farmers in SSA and have associated crop losses and contaminations by mycotoxins [8, 69, 70]. Some other traditional methods include the use of closed containers, which may still favor the proliferation of mycotoxin-producing fungi. The use of sacks (such as sisal bags and gunny bags) and granaries can also encourage attacks by rodent pests (rats) and insect pests (weevils) [71]. The use of advanced storage facilities such as cement or metal bins is considerably effective and durable. However, due to their high initial cost, it is not affordable to most small-scale stakeholders hence challenging to encourage them to adopt these innovative storage methods [67]. The use of PICS bags can substitute metal silos because they are effective against rats, are less costly, and can reduce aflatoxin contamination, substantially. Kamano et al. [48] showed that proper storage in airtight structures such as hermetic bags reduced aflatoxin contamination. However, in Kenya, storage of maize is mostly done in gunny bags [37, 48]. This study finds that maize grains are mainly packed in gunny bags and least packed in sisal bags, PICS bags, and hermetic bags. Compared to maize grains stored in hermetic structures (metallic bins and PICS bags), the levels of aflatoxin-related fungi are most likely to be found in grains stored in polypropylene (gunny) bags [70]. The use of sacks (such as sisal bags and gunny bags) and granaries can encourage attacks by rodent pests (rats) and insect pests (weevils) making the grains more vulnerable to fungal attacks [71], findings that were also confirmed by Kamano et al. [37].

Consumers showed strong and positive attitudes toward good quality and aflatoxin-safe maize grains that they purchased from the markets. They also agreed that mould in maize grain stores can pose a risk of aflatoxin contamination; learning about aflatoxin safety through training is important; and when aflatoxin maize grains are well cooked, they will not always be safe to consume. The majority of vendors trust that the maize grains they sell are without observable characteristics of aflatoxin (i.e., broken grains/kernels, mouldy grains/kernels, and safe from aflatoxin contamination). However, the consumers disagreed with these beliefs indicating the biases among the vendors on the quality of maize. Consumers trust that they have minimal chances of purchasing maize containing aflatoxin.

## 5. Conclusions

Aflatoxin contamination in maize grain is a global food safety concern as it reduces the quality of the maize grain and has adverse health effects on humans. Supplying or purchasing maize grains that are free from aflatoxin contamination must be highly prioritized by vendors and consumers. Based on the findings, awareness of the danger of aflatoxin contamination was average among vendors (48.2%) and consumers (52.1%). The level of KAPs on aflatoxin contamination of maize grains varied by subcounty, education level, gender, and age. Generally, there were adequate knowledge, appropriate practices, and good attitudes. However, there is a need to associate the vendors' and consumers' sociodemographic factors and KAPs with the level of aflatoxin contamination in the maize grains being traded through rounds of physical examination. This information is necessary to help in training the stakeholders (vendors and consumers) in the informal markets on the correlation between observable maize grain quality attributes, which is a cheap and easy method of identifying aflatoxin contamination, and unobservable attributes (mycotoxins especially aflatoxin), which is an expensive and unaffordable method.

The analysis of KAPs acquired from respondents revealed that the less educated respondents were less aware of the health risks associated with eating maize grains contaminated with aflatoxin than the more educated respondents. This shows that education is the most effective way to lower the risk of consuming or selling aflatoxin-contaminated maize grains. Therefore, further research is needed to determine the prevalence and effects of aflatoxin in the maize grains sold in informal marketplaces and to develop management methods to lower health hazards. Adopting appropriate storage techniques, such as using upgraded granaries and hermetic bags, is essential to avoid aflatoxin incidence during maize storage. Preventing damage to maize grains from insects and rodents is also an effective measure of controlling aflatoxin contamination. Sorting the maize grains to remove broken, contaminated, and discolored grains before storing them reduces the exacerbation of aflatoxin contamination.

## Figures and Tables

**Table 1:** Estimated population and sample size of vendors in small- to medium-sized informal markets.

**Subcounties in Meru County**	**Population (** **N** **)**	**Sample size (** **n** **)**
Buuri	121	65
Igembe Central	143	71
Igembe North	9	8
Igembe South	33	27
Imenti Central	87	54
Imenti North	57	41
Imenti South	400	103
Tigania East	53	39
Tigania West	64	44

**Table 2:** Question categorization and correctness of the answers based on the knowledge of vendors and consumers on aflatoxin.

**Questions**	**Answers categorized in binary correct scores (** **y** **e** **s** = 1, **no** = 0**)**
Maize vendors	
Have you ever heard of aflatoxin before today?	1 = yes, 0 = no
Do you know any health effects that come from eating aflatoxin-contaminated maize grains?	1 = stomach pain, diarrhea, cancer, death, increased vulnerability to diseases, bloating, headaches, jaundice, memory loss, vomiting; 0 = any another answer
Maize consumers	
Have you ever heard of aflatoxin before today?	1 = yes, 0 = no
Do you know any health effects that come from eating aflatoxin-contaminated maize grains?	1 = stomach pain, diarrhea, cancer, death, increased vulnerability to diseases, bloating, headaches, jaundice, memory loss, vomiting; 0 = any another answer
Are you able to tell if maize contains aflatoxin by visual inspection?	1 = yes, 0 = no

**Table 3:** Question categorization and correctness of the answers based on the practices and attitudes of vendors and consumers on aflatoxin.

**Questions**	**Answers categorized in binary correct scores (** **y** **e** **s** = 1, **no** = 0**)**
Maize consumers	
Do you normally check the quality of maize when purchasing maize for sale in this market?	1 = yes, 0 = no
Is proper storage of maize at the shop/kiosk by the sellers important in reducing contamination of maize with aflatoxins?	1 = strongly agree, agree; NA = neither agree nor disagree; 0 = disagree, strongly disagree
Can poor management of grains during transport from the farm to the market accelerate or expose maize to aflatoxin contamination?	1 = strongly agree, agree; NA = neither agree nor disagree; 0 = disagree, strongly disagree
Does sorting maize at the shop/market improve the quality of maize in terms of aflatoxin contamination?	1 = strongly agree, agree; NA = neither agree nor disagree; 0 = disagree, strongly disagree
Maize consumers	
Do you check maize quality during the purchase?	1 = yes, 0 = no
Do you have different uses based on the quality of maize?	1 = yes, 0 = no
Is it your responsibility as a consumer to make sure that the maize you purchase is of good quality?	1 = strongly agree, agree; NA = neither agree nor disagree; 0 = disagree, strongly disagree
Is it your responsibility as a consumer to make sure that the maize you purchase is safe from aflatoxin contamination?	1 = strongly agree, agree; NA = neither agree nor disagree; 0 = disagree, strongly disagree
Is it important to learn about aflatoxin safety through training?	1 = strongly agree, agree; NA = neither agree nor disagree; 0 = disagree, strongly disagree
When aflatoxin maize grain is well cooked, is it always safe to eat?	0 = strongly agree, agree; NA = neither agree nor disagree; 1 = disagree, strongly disagree
Will mould in maize grain stores not pause any risk of aflatoxin infection?	0 = strongly agree, agree; NA = neither agree nor disagree; 1 = disagree, strongly disagree
Should maize grain kernels be maintained under good storage conditions?	1 = strongly agree, agree; NA = neither agree nor disagree; 0 = disagree, strongly disagree

**Table 4:** Sociodemographic characteristics of respondents.

**Variables**	**Vendors**		**Consumers**	
	**Frequency (** **N** = 452**)**	**Percentage (%)**	**Frequency (** **N** = 86**)**	**Percentage (%)**
Age (years)				
18–29	71	16.5	22	25.6
30–39	115	26.7	24	27.9
40–49	131	30.5	17	19.8
50–59	58	13.5	14	16.3
> 60	55	12.8	9	10.5
Gender				
Female	374	82.7	72	83.7
Male	78	17.3	14	16.3
Educational status				
No formal education	19	4.3	5	5.8
Primary level	208	47.1	46	53.5
Secondary level	158	35.8	25	29.1
Tertiary level	57	12.9	10	11.6
Maize farmer				
Yes	—	—	73	84.9
No	—	—	13	15.1
Type of vendor				
Kiosk/shop owner	341	75.4	—	—
Open air/“Kibanda”	111	24.6	—	—
Subcounties				
Buuri	65	14.4	10	11.2
Igembe Central	71	15.7	14	15.7
Igembe North	8	1.8	8	9.0
Igembe South	27	6.0	10	11.2
Imenti Central	54	12.0	9	10.1
Imenti North	41	9.1	7	7.9
Imenti South	103	22.8	10	11.2
Tigania East	39	8.6	11	12.4
Tigania West	44	9.7	10	11.2

**Table 5:** Knowledge of aflatoxin contamination among the vendors and consumers of maize grains.

**Questions capturing the knowledge of respondents**	**Vendors**		**Consumers**	
	**Frequency (** **n** **)**	**Percentage (%)**	**Frequency (** **n** **)**	**Percentage (%)**
Have you ever heard of aflatoxin before today?				
Yes	388^a^	86.8	61^a^	70.9
No	59^b^	13.2	25^b^	29.1
What are the health effects of consuming aflatoxin-contaminated maize?				
Stomach pain	235^a^	48.2	50^a^	52.1
Cancer	78^b^	16.0	13^b^	13.5
Death	61b^c^	12.5	6^c^	6.3
Diarrhea	57b^c^	11.7	15^b^	15.6
Increases vulnerability to disease generally	36^c^	7.3	7^c^	7.3
Liver failure/jaundice	11^d^	2.3	1^c^	1.0
Vomiting	6^d^	1.2	1^c^	1.0
Bloating	2^d^	0.4	2^c^	2.1
Headache	2^d^	0.4	1^c^	1.0
Are you able to tell if maize contains aflatoxin by visual inspection?				
Yes	—	—	63^a^	76.8
No	—	—	19^b^	23.2
If yes, what are the signs that maize contains unsafe levels of aflatoxins?				
Amount of mouldy grains	—	—	48^a^	50.5
Discoloration	—	—	31^ab^	32.6
Moisture levels	—	—	7^c^	7.4
Amount of foreign matter/dirt in maize	—	—	5^c^	5.3
Amount of broken kernels	—	—	2^c^	2.1
Presence of insects			2^c^	2.1

*Note:* The same letters within columns indicate no statistical difference at *p* > 0.05 (the Tukey post hoc test).

**Table 6:** Maize grain quality dimensions assessed by the vendors and consumers.

**Questions**	**Vendors**		**Consumers**	
	**Frequency (** **n** **)**	**Percentage (%)**	**Frequency (** **n** **)**	**Percentage (%)**
Do you check maize quality during purchase?				
Yes	430^a^	96.2	82^a^	95.3
No	17^b^	3.8	4^b^	4.7
Quality dimensions assessed				
The proportion of visibly mouldy grains	260^a^	25.5	45^a^	29.0
Moisture level	206^ab^	20.2	20^b^	12.9
Proportion of foreign matter in the maize	136^bc^	13.3	22^b^	14.2
Size of the grain	116^cd^	11.4	23^b^	14.8
Proportion of broken maize kernels	89^de^	8.7	19^b^	12.3
Insect infestation	70^de^	6.9	8^c^	5.2
Aflatoxin levels	63^de^	6.2	5^c^	3.2
Color change	48^e^	4.7	—	—
Presence of pesticides	17^f^	1.7	6^c^	3.9
Maize variety	10^fg^	1.0	5^c^	3.2
Weight of maize	3^g^	0.3	2^c^	1.3
If the moisture level is selected, the method used to check				
Use of hands (sequencing)	145^a^	46.3	11^a^	57.9
Biting	128^ab^	40.9	8^b^	42.1
The sound produced while shaking	13^c^	4.2	—	0.0
Visual inspection	10^c^	3.2	—	—
Smelling of maize grains	6^c^	1.9	—	—
Moisture meter	4^c^	1.3	0^c^	0.0
The temperature when in the sack	4^c^	1.3	—	—
Weight of grains	2^c^	0.6	—	—
Mouldy content	1^c^	0.3	—	—
If aflatoxin is selected, what test is used?				
Proportion of mouldy grains	61^a^	93.8	6a	75.0
Moisture levels (well-dried maize)	0^b^	0.0	1^b^	12.5
Proportion of broken kernels in the maize	0^b^	0.0	1^b^	12.5
Proportion of dirt/foreign matter in the maize	0^b^	0.0	0^b^	0.0
Smell of grains	1^b^	1.5	—	—
Rapid test	2^b^	3.1	0^b^	0.0
Take samples to a laboratory	1^b^	1.5	0^b^	0.0
Do you normally mix maize of different qualities to improve the average quality?				
Yes	75^b^	16.8	—	—
No	372^a^	83.2	—	—
How do you normally use the maize that you purchase in the market?				
“Githeri”	—	—	56^a^	36.4
Milling for flour	—	—	55^a^	35.7
“Muthokoi”	—	—	34^ab^	22.1
Livestock feed	—	—	3^c^	1.9
Porridge	—	—	3^c^	1.9
“Njenga”	—	—	2^c^	1.3
“Muthikore”	—	—	1^c^	0.6

*Note:* Different superscripts indicate statistical differences within the column.

**Table 7:** Vendors' and consumers' attitudes toward aflatoxin contamination in maize grains.

**Statements to capture respondents' attitude**	**Vendors**		**Consumers**	
	**Freq. (** **n** **)**	**%**	**Freq. (** **n** **)**	**%**
The maize sold in the market is on many occasions without any broken grains/kernels.				
Strongly agree	298^a^	66.7	2^c^	2.3
Agree	109^b^	24.4	16^b^	18.6
Neither agree nor disagree	10^cd^	2.2	5^c^	5.8
Disagree	6^d^	1.3	30^a^	34.9
Strongly disagree	24^c^	5.4	32^a^	37.2
The maize sold in the market is on many occasions without any mouldy grains/kernels.				
Strongly agree	256^a^	57.3	3^c^	3.5
Agree	131^b^	29.3	11^c^	12.8
Neither agree nor disagree	17^c^	3.8	6^c^	7.0
Disagree	16^c^	3.6	22^b^	25.6
Strongly disagree	27^c^	6.0	44^a^	51.2
The maize sold in the market is on many occasions safe from aflatoxin contamination.				
Strongly agree	184^a^	41.2	3^c^	3.5
Agree	157^a^	35.1	5^c^	5.9
Neither agree nor disagree	18^c^	4.0	8^c^	9.4
Disagree	31^b^	6.9	24^b^	28.2
Strongly disagree	57^b^	12.8	45^a^	52.9
How many consumers do you believe will have maize containing aflatoxin, out of 10?				
0/10	—	—	36^a^	41.9
1/10	—	—	10^b^	11.6
2/10	—	—	12^b^	14.0
3/10	—	—	11^b^	12.8
4/10	—	—	1^c^	1.2
5/10	—	—	6^c^	7.0
6/10	—	—	2^c^	2.3
7/10	—	—	1^c^	1.2
8/10	—	—	1^c^	1.2
9/10	—	—	1^c^	1.2
10/10	—	—	5^c^	5.8

*Note:* Different superscripts indicate statistical differences within the column.

**Table 8:** Packaging and storage practices among maize grain vendors.

**Practice of respondents to reduce fungal infection**	**Frequency (** **n** **)**	**Percentage (%)**
How is maize packaged for storage, using?		
Gunny bags	384^a^	87.3
Sisal bags	50^b^	11.4
Hermetic bags	6^c^	1.4
How is the maize stored in the storage area?		
On concrete floor	384^a^	83.8
On the ground but on top of a plastic sheet	50^b^	10.9
On the ground but on top of a gunny bag	11^c^	2.4
On bare ground	6^c^	1.3
Palm basket (“Kikapu”)	4^c^	0.9
Plastic buckets	3^c^	0.7

*Note:* Different superscripts indicate statistical differences within the column.

**Table 9:** Practices toward minimizing aflatoxin contamination in maize grain among consumers.

**Practice of respondents to reduce aflatoxin contamination**	**Always**	**Often**	**Sometimes**	**Rarely**	**Never**
Maize grain kernels are maintained under good storage conditions	71 (82.6)^a^	4 (4.7)^bc^	9 (10.5)^b^	2 (2.3)^bc^	0 (0.0)^a^
Maize grain kernels are stored using					
Gunny bags	55 (64.0)^a^	7 (8.1)^b^	14 (16.3)^b^	2 (2.3)^b^	8 (9.3)^b^
PICS bags	9 (10.5)^b^	3 (3.5)^c^	8 (9.3)^bc^	16 (18.6)^b^	50 (58.1)^a^
Hermetic bags	13 (15.1)^b^	11 (12.8)^b^	10 (11.6)^b^	15 (17.4)^b^	36 (41.9)^a^
Drying of high moisture content maize grain is carried out					
On the ground with canvas	69 (80.2)^a^	8 (9.3)^b^	4 (4.7)^b^	4 (4.7)^b^	1 (1.2)^b^
On the ground without canvas	0 (0.0)^b^	2 (2.3)^b^	2 (2.3)^b^	5 (5.8)^b^	77 (89.5)^a^
In an open store	30 (34.9)^a^	3 (3.5)^b^	12 (14.0)^bc^	7 (8.1)^b^	34 (39.5)^a^

*Note:* Different superscripts indicate statistical differences within the column.

## Data Availability

All the data are presented in this paper. However, the data generated or analyzed during this study are available from the corresponding author upon reasonable request.
